# *In-silico* analysis of *Bifidobacterium bifidum* strain 900791 genome in the context of the *B. bifidum* pangenome

**DOI:** 10.3389/fcimb.2026.1744409

**Published:** 2026-07-01

**Authors:** Juan P. Cárdenas, Boris Vidal-Veuthey, Kevin Meza, Daniel E. Almonacid, Anastasia Gutkevich

**Affiliations:** 1 Departamento de Ciencias Biológicas, Facultad Ciencias de la Vida, Universidad Andres Bello, Santiago, Chile; 2 Centro de Genómica y Bioinformática, Facultad de Ciencias, Ingeniería y Tecnología, Universidad Mayor, Santiago, Chile; 3 Programa de Doctorado en Genómica Integrativa, Facultad de Ciencias, Ingeniería y Tecnología, Universidad Mayor, Santiago, Chile; 4 R & D Department, Bifidice SpA, Santiago, Chile; 5 Eigenify Inc., San Francisco, CA, United States

**Keywords:** Bifidobacterium bifidum, pangenome, probiotic traits, probiotics, whole genome sequencing

## Abstract

**Introduction:**

Bifidobacterium bifidum is a key member of the human gut microbiota with well-recognised roles in intestinal homeostasis, glycan metabolism, and immunomodulation. Strain 900791, isolated from the meconium of a Siberian infant, has been used as a commercial probiotic ingredient for decades and has demonstrated, in previously published clinical trials, an ability to improve lactose tolerance and reduce gastrointestinal symptoms in both children and adults; however, the genomic basis for these properties has not been characterised.

**Methods:**

We present the complete genome sequence and comprehensive in silico genomic and pangenomic analysis of B. bifidum strain 900791. Hybrid sequencing was used for genome assembly. Phylogenomic analysis, including core genome MLST (cgMLST), integrated 229 high-quality B. bifidum genomes, representing the largest dataset for this species to date. Functional annotation and carbohydrate-active enzyme (CAZyme) profiling, antimicrobial resistance prediction, and bioinformatic screening for probiotic-associated genomic features were also performed.

**Results:**

Hybrid sequencing yielded a single circular chromosome of 2,280,092 bp, comprising 1,852 protein-coding sequences. Phylogenomic analysis revealed that strain 900791 belongs to a clonal subgroup of nine closely related strains (>99% cgMLST identity), consistent with a geographically structured lineage. The species pangenome comprised 4,152 orthogroups and a core of 1,450 gene families; 23 orthogroups were exclusive to the 900791 clonal subgroup, including a predicted lantibiotic biosynthetic cluster. CAZyme profiling identified glycoside hydrolase families associated with human milk oligosaccharide degradation (GH2, GH20, GH33, GH84), mucin glycan cleavage (including ten GH families), and lactose metabolism (GH2, GH42). Safety assessment identified only species-typical resistance to mupirocin and rifampicin, with no acquired resistance markers. Bioinformatic screening of the clonal subgroup detected the presence of adhesion-associated proteins, acid resistance systems, bile salt tolerance determinants, oxidative stress response proteins, and two putative bacteriocin gene clusters.

**Discussion:**

These findings provide a genomic framework consistent with the documented clinical role of strain 900791 in lactose tolerance and support its further investigation as a candidate probiotic. The probiotic-associated features identified here may help explain its observed properties and represent priority targets for experimental validation in future in vitro and in vivo studies.

## 1 Introduction

*Bifidobacterium bifidum* is a key member of the human gastrointestinal microbiota, recognized for its primary roles in maintaining gut homeostasis, modulating immune responses, and shaping the microbial ecosystem, especially during human early life (Turroni et al., 2019). Several strains of this bacterium have been extensively used in the production of various foods such as cultured milk and other dairy products (Yaeshima et al., 1996; Gomi et al., 2018). *B. bifidum* strains have been also utilized in therapeutic preparations for the treatment of digestive disturbances in infants and adults (Totsu et al., 2014; Guglielmetti et al., 2011; Gomi et al., 2018). *B. bifidum* exhibits the metabolic capacity to degrade host-derived glycans, including human milk oligosaccharides (HMOs) during its colonization in infants, as well as mucin glycans in the adult gut (Xiao et al., 2024). This feature enables this organism to colonize and persist throughout different human life stages. *B. bifidum* supports the integrity of the intestinal epithelial barrier by stimulating mucin production and enhancing the protective mucus layer that limits pathogen invasion and maintains intestinal integrity (Martín et al., 2020; Xiao et al., 2024). Additionally, the ability of *B. bifidum* to degrade different glycans not only sustains its own growth but also facilitates cross-feeding interactions with other gut microbes (Xiao et al., 2024). This syntrophy promotes an eubiotic microbial community and increases the production of short-chain fatty acids (SCFAs), such as acetate and butyrate, which are considered beneficial substrates for gut health and epithelial function (Morrison and Preston, 2016; Parada Venegas et al., 2019). *B. bifidum* strains also were reported to produce indole-3-lactic acid, a metabolite involved in immunomodulation, with a potential psychobiotic effect (Qian et al., 2024). Moreover, *B. bifidum* strains can also stimulate mucin synthesis in an indirect fashion, strengthening the mucus barrier and reducing intestinal permeability, which is crucial for protecting the host from pathogenic challenges (Gutierrez et al., 2023; Engevik et al., 2019). Different studies suggested that *B. bifidum*, often in combination with other bacteria, can also influence the gut microbial community by fostering the growth of beneficial microbes while suppressing inflammation and potential pathogens (Shin et al., 2024). Its presence has also been linked to improved bowel function and relief from constipation, possibly through modulation of gut neurotransmitters (Fuyuki et al., 2021; Makizaki et al., 2021). In addition to its metabolic activity, *B. bifidum* exerts significant immunomodulatory effects: some studies suggest that this organism interacts with intestinal epithelial and immune cells, such as dendritic cells, to promote anti-inflammatory responses and support the maturation of the immune system, particularly during the early years (Lim and Shin, 2020). These interactions help maintain immune tolerance and prevent excessive gut inflammation, contributing to overall host health. The main sources of *B. bifidum* isolation reflect its ecological niche and metabolic specialization. It is predominantly isolated from the feces of breast-fed infants (Shah, 2022) or even breast milk (Martín et al., 2009). HMOs in human breast milk are pivotal substrates to sustain *B. bifidum* growth facilitating vertical bacterial transmission from mother to infant and establishing early colonization of the infant gut (Milani et al., 2015); therefore, *B. bifidum* affinity for those oligosaccharides can also be targeted for isolation or enrichment (Gotoh et al., 2018). The rise of genomics, and more recently, the emergence of Next-Generation Sequencing (NGS) technologies, made a tremendous impact on microbiology, reflected in the generation and availability of massive amounts of genomic data (Satam et al., 2023). Nowadays, whole genome sequencing (WGS), very useful for the generation of a first landscape of main metabolic and functional features for a bacterial strain, has been facilitated with hybrid sequencing techniques, combining the virtues and properties of short- (e.g., Illumina, DNBSEQ) and long-read (e.g., Nanopore, Pacbio) sequencing (Neal-McKinney et al., 2021). *B. bifidum* is just one of the several *Bifidobacterium* species, sequenced from a variety of different hosts, sources or geographical contexts (Alessandri et al., 2021). Recent genomic analyses of *Bifidobacterium bifidum* have elucidated its evolutionary strategies for gut colonization, emphasizing pan-genome plasticity and niche-specific adaptations. The first report for a genomic analysis for *B. bifidum* (the PRL2010 strain) showed the presence of genes encoding potential enzymes for mucin degradation such as extracellular sialidases (GH33), fucosidases (GH29), and lacto-N-biosidases (GH20) to degrade mucin oligosaccharides, enabling it to exploit mucin as a primary carbon source, confirming this feature by transcriptomic profiling (Turroni et al., 2010). Age-related genomic and phenotypic variation in *B. bifidum* was also explored: the comparison of six strains isolated from subjects in different age ranges suggested enhanced genomic capacity for utilizing 6’-sialyllactose, a key component of HMOs, in strains from children and young people (0–17 years old) in comparison with adults (18–65 years old) (Wei et al., 2023). Another comparative genomics study (Abdelhamid and El-Dougdoug, 2021) analyzed 15 *B. bifidum* genomes from diverse human niches such as gut, vagina, and breast milk; this study showed the presence of differentially conserved carbohydrate active enzymes (CAZymes) reflecting different adaptation across different niches. Another recent study (Lu et al., 2021) compared 140 strains isolated from Chinese adults and infants, revealing an open pan-genome of 8, 399 genes and a conserved core genome (638 genes). Phylogenetic clustering by geographical origin and isolation source (infant vs. adult feces) highlighted the influence of host ecology on genetic divergence. Genetic diversity was pronounced in carbohydrate metabolism pathways, including glycoside hydrolases (GHs) targeting host-derived mucins, and antimicrobial competition factors like bacteriocin operons. Two more recent studies (Li et al., 2024a, 2024b) improved the current landscape of the role of *B. bifidum* in the human microbiome.

Among several *B. bifidum* strains, an interesting case corresponds to the 900791 strain (formerly known as *B. bifidum* 1), isolated from the meconium of an infant from Siberia (Gutkevich et al., 2003 ). An early study demonstrated that this strain could be used in a probiotic ice-cream formulation (in a dose of 10^5^ CFU/g) and, in combination with a strain and *L. plantarum* (10^6^ CFU/g) could improve the relative abundance of fecal bifidobacteria and decrease the abundance of fecal pathogenic and opportunistic microorganisms in a cohort of 7, 939 participants (6, 854 of them, children aged 2 to 7 years) (Gutkevich et al., 2003). More evidence indicates that 900791 exert probiotic properties: another previous pilot clinical trial, conducted in 50 children (7–11 years old) with chronic or functional gastrointestinal disorders, showed that the use 5 x 10^7^ CFU of this strain and 0.2 g of lactose led to a decrease in the frequency and severity of respiratory infections, an improvement in overall morbidity indicators, and good tolerance were observed with prolonged use (Meskina et al., 2018). Another study, conducted with 50 adults subjects, utilized a ice-cream preparation containing 10^5^ CFU/g and 10^7^ CFU/g *B. bifidum* 900791 to evaluate its role in amelioration of hypolactasia/lactose intolerance (Aguilera et al., 2021); this intervention significantly improved the control of symptoms such as flatulence, pain, and diarrhea, and the concentration of hydrogen in exhaled air, even at 10^5^ CFU/g. Therefore, strain 900791 has been used repeatedly in the probiotic industry for years, as part of infant formulas and food products. The creation of “bio-ice cream” has included strains of the *B. bifidum* species, which have been used for the prevention of infectious diseases (Gorelov et al., 2005).

Strain 900791, despite its verified probiotic properties, remains to be characterized at the genomic level. Comparative genomics offers a powerful framework to predict functional capabilities and better understand these properties. Previous comparative studies for *B. bifidum* have relied on datasets from 15 (Abdelhamid and El-Dougdoug, 2021) to 140 assemblies (Lu et al., 2021); additionally, most comparative studies have focused into general features, instead of strain- or cluster-specific properties. Here, we present an *in-silico* genome analysis of *B. bifidum* strain 900791 in the context of the pangenome of this species, based on filtered dataset of 229 high-quality genomes, analyzed by a graph-based pangenome pipeline, representing the largest *B. bifidum* pangenomic dataset published to date. Within this context, we specifically screen for previously reported probiotic-associated traits and predict genomic features that distinguish strain 900791 from its closest relatives, including clonal group-specific genes and other genomic characteristics that may underlie its probiotic potential.

## 2 Methodology

### 2.1 DNA isolation, library preparation, hybrid sequencing and basic annotation

The genomic DNA of strain 900791 was extracted using the *FastDNA SPIN Kit for Soil* (MP Biomedicals) in accordance with the manufacturer’s instructions. The DNA was quantified using gel electrophoresis with 1% of agarose gel and fluorescence-based Qubit dsDNA DNA quantification System (ThermoFisher, USA). Hybrid sequencing of the isolate was carried out at EzBiome (Gaithersburg, MD, USA). Briefly, the genomic DNA was sequenced on an Illumina NextSeq2000 (2 × 150 bp) and an R10.4.1 flow cell of a Nanopore PromethION (Eugene, USA). The Illumina library was prepared using the NEBNext^®^ Ultra™ II FS DNA library kit for Illumina, while the Nanopore library was prepared using v14 library prep chemistry without fragmentation or size selection. The resulting sequencing reads were filtered using Filtlong v0.2.1 (parameters: “--min_length 1000 --keep_percent 95”, see https://github.com/rrwick/Filtlong) by removing the 5% worst fastq reads. Nanopore reads were then assembled with Flye v2.9.2 (Kolmogorov et al., 2019). Illumina reads were aligned to the draft assemblies using BWA with the ‘-a’ flag (Li, 2013). Alignment files and draft assemblies were used to produce polished assemblies, using Polypolish v0.5.0 (Wick and Holt, 2022). Polished assemblies were checked for contamination using CheckM2 v1.1 (Chklovski et al., 2023), annotated with Prokka v1.14.6 (Seemann, 2014) and circular genome maps were produced with GenoVi v0.4.3 using default parameters (Cumsille et al., 2023).

### 2.2 Antibiotic resistance gene profiling

Antibiotic resistance gene profiles were evaluated using three different approaches. The first consisted in the use of a pre-built *bowtie2* (Langmead and Salzberg, 2012) database composed of NCBI’s *National Database of Antibiotic-Resistant Organisms* (NDARO, www.ncbi.nlm.nih.gov/pathogens/antimicrobial-resistance/) reference genes. The read dataset of the 900791 strain was mapped against these genes using *bowtie2* v2.5.4 with the “--very-sensitive” option, and the output was then converted and sorted by *samtools* v1.21 (Li et al., 2009). Finally, for each gene found, depth and coverage were calculated by using samtool’s *mpileup* script. The second approach consisted in analyzing the genome assembly by using ResFinder v4.7.2 (Florensa et al., 2022) using a cutoff of 90% identity and 60% minimum coverage. The third approach utilized the predicted coding sequences (CDS) repertoire, which was analyzed by the *Resistance Gene Identifier* tool from the CARD database (RGI-main v6.0.3, CARD v4.0.0), considering perfect and strict hits only (Alcock et al., 2023).

### 2.3 Selection of *B. bifidum* genomes and generation of the phylogenomic tree

The following method differs slightly from those presented in a previous study (González et al., 2023). All genomes available in NCBI Genomes belonging to any taxonomic group associated to *Bifidobacterium* (NCBI:taxid 1678) were downloaded from the NCBI Genbank FTP site in early March 2025, and their metadata was extracted from their respective NCBI Project/Biosample accession (unless more curation were required). The taxonomic identity was confirmed by using the program ‘classify_wf’ of the GTDB-Tk program, version 2.4.0 (Chaumeil et al., 2022), using the database release 220 as the reference, selecting all genomes classified into the *B. bifidum* species (“s:__Bifidobacterium bifidum”). Genome completeness and contamination were calculated using the program ‘predict’ from CheckM2 v1.1 (see above). Only genomes with the aforementioned taxonomic assignment, plus a completeness higher than 90%, contamination lower than 5% and an assembly N50 value equal or higher than 200 Kbp (see Results) were selected. The CDS of each resulting genome set was predicted using Prodigal, version 2.6.3 (Hyatt et al., 2010) (relevant parameters: *-q -c -m*). Using this CDS dataset (in addition to the 900791 strain), the orthogroup catalog was calculated by Orthofinder version 2.5.5 (Emms and Kelly, 2019) using “-S diamond_ultra_sens -y -og” as relevant parameters.

To make the phylogenomic tree, two parallel strategies were implemented. The first approach was the use of the protein sequence 769 concatenated, single-copy conserved orthogroups (defined by Orthofinder): a multiple sequence alignment was constructed from those orthogroups by using MAFFT version 7.490 (parameters: --maxiterate 1000 --localpair) (Katoh et al., 2002); the alignment was used by *iqtree* version 2.1.4 (parameters: -m TEST --alrt 1000) to generate a maximum likelihood-based tree with an aLRT with 1000 replicates as the branch support test (Minh et al., 2020). The RefSeq CDS set from *Bifidobacterium longum* subsp. *longum* JCM 1217 (GCF_000196555.1) was utilized as the outgroup by adding its CDS repertoire to the previous orthogroup catalog with the “-b” and “-f” parameters in Orthofinder, and adding the aligned orthologs to the previously calculated single-copy core multiple sequence alignment. The second approach, without outgroup, utilized the MAFFT-aligned nucleotide sequences of 1,450 core gene families defined by Panaroo v1.6.0 (Tonkin-Hill et al., 2020) created by the script “panaroo-msa” of the Panaroo package (relevant parameter: “--codons”). The phylogenomic tree was visualized using the *ggtree* R package (Xu et al., 2022). A visual comparison of the two trees (i.e., tanglegram) was computed using the commands “cophylo” and “plot” of the *phytools* R package v2.5-2 (Revell, 2024). The comparison of the topologies between the amino acid- and nucleotide-based phylogenies was performed using the Robinson-Foulds (RF), and the Kuhner-Felsenstein (KF) distances, using the *phangorn* R package v2.12.1 (Schliep, 2011).

### 2.4 Pangenome analysis

The calculation of the pangenome was made by Panaroo v1.6.0 ( (Tonkin-Hill et al., 2020), using the following relevant parameters: “–clean-mode strict –remove-invalid-genes –aligner mafft –core_threshold 0.95 –family_threshold 0.7 –threshold 0.95”. The orthogroup matrix, obtained from Panaroo results, was utilized for different pangenome metrics. Pangenome curves were created using the *panplots* function in R (created by *SioStef*, https://github.com/SioStef/panplots/), using 1000 permutations. The gamma value from the Power law deduced from the pangenome curve (Tettelin et al., 2008) was calculated using the function *curve_fit* from *scipy* python package, using the equation *“y = ax^γ^”*, applied on the *panplots* output. Alternatively, to calculate the alpha value from the Heap’s Law equation (Tettelin et al., 2008), the Panaroo matrix was used by the command “*heaps*” from the *micropan* R package v2.1 using 1,000 permutations (Snipen and Liland, 2015). Heatmaps were created by using the *pheatmap* function from R. Figures were elaborated with *ggplot2* and the *ggarrangment* packages. In order to create core-genome multilocus sequence typing (cgMLST), the nucleotide sequences for the single-copy gene families were retrieved and an allele database was created by using pyMLST (Biguenet et al., 2023). The dataset of 228 genomes plus the 900791 strain were cataloged using this dataset.

### 2.5 Additional functional annotation processes

The predicted CDS repertoire from both the 900791 strain genome and the selected available *B. bifidum* genomes were analyzed by EggNOG-mapper v2.1.12 (parameters: “--tax_scope_mode narrowest --tax_scope prokaryota_broad --go_evidence experimental”, see (Cantalapiedra et al., 2021)). Metabolic pathway prediction was conducted by the combination of the KAAS (Moriya et al., 2007), BlastKOALA (Kanehisa et al., 2016), and KofamKOALA (Aramaki et al., 2020) tools. KAAS was utilized in best-bidirectional-hit (BBH) mode, using *Prokaryote* set as reference; BlastKOALA was used also with the *Prokaryote* reference set; KofamKOALA was configured using 10^–6^ as the threshold E-value. Results were mapped by using KEGG mapper. Carbohydrate active enzymes (CAZymes) (Flint et al., 2012) were searched using the HMM database v11 from dbCAN, the search tool based on CAZy (Zheng et al., 2023), using an e-value < 1e-10. Bacteriocins were predicted by using the BAGEL4 server (van Heel et al., 2018) using the assembly data. Subcellular localization predictions using the 900791 strain CDSs were performed by PSORTb v3.0 for Gram-positive bacteria (Yu et al., 2010). When necessary, Diamond v2.1.8 (Buchfink et al., 2015) was used for BLASTP searches of the predicted CDSs of the 900791 strain and other genomes against the Swissprot database (Release 2025_03).

## 3 Results

### 3.1 Overall properties of the genome of *Bifidobacterium bifidum* strain 900791

The hybrid sequencing of the genome of the *B. bifidum* 900791 strain resulted in a complete genome (**Supplementary Figure 1**), consisting of a single circular chromosome of 2, 280, 092 base pairs (average depth: 22x) with an average G+C content of 62.43%. The taxonomic status of this genome was confirmed by two approaches: in the first approach, the predicted 16S rRNA gene from this genome exhibited 100% identity with the 16S rRNA gene from *B. bifidum* strains KCTC3202 and NBRC100015 (accessions NR_117505.1 and NR_113873.1 respectively); in the second approach, the identity of this genome as *B. bifidum* was confirmed by ANI against the genome of *B. bifidum* ATCC 29521 using GTDBtk (GCF_001025135.1, ANI = 98.86%). Prokka annotation of this genome revealed 1, 852 protein-coding genes), 53 tRNA genes, and 6 rRNA genes. The protein-coding sequences account for approximately 85% of the total genome. This genome contained no plasmids. According to CheckM2, its contamination level is 0.12% (Specific model).

From the CDS repertoire of this strain, some general features were predicted. Using PSORTb as the predictor of subcellular localization of the proteins, the analysis of the CDS repertoire of *B. bifidum* 900791 (**Supplementary Figure 2A**) detected a set of 28.2% proteins outside the cytoplasm/cytosol, predicted to be located in the plasmatic membrane (n = 476, % 25.7), or the cell wall (n = 18, % 0.97); a total of 28 CDS (% 1.51) were predicted to be secreted into extracellular medium. This may suggest that the genetic repertoire of this species may include potentially secreted proteins that deserve further attention. The functional annotation results from the EGGNOG mapper database (excluding 257 unclassified CDS) showed that the proportion of sequences associated with *metabolism* (categories C, G, E, F, H, I, P, Q) represents 36.78% of the total assignments for this CDS repertoire (**Supplementary Figure 2B**); functions associated with *information storage and processing* (categories J, A, K, L) represented 26.43% of all the CDSs, whereas functions associated with *cellular processes and signaling* (categories D, Y, V, T, M, N, Z, W, U, O, X) represented 19.3% of CDSs. Sequences from the metacategory *poorly characterized* (categories R and S) represented 17.49% combined. Analyzing by categories (**Supplementary Figure 2C**), the most represented in the 900791 strain were associated with category S (“*Function unknown*”, with 17.49% of assignments), followed by genes involved in *Amino acid transport and metabolism* (category E, with 9.18% assignments), *Replication, recombination and repair* (category L, 9.01%), *Translation, ribosomal structure and biogenesis* (J, 8.77%), *Transcription* (K, 8.6%) and *Carbohydrate transport and metabolism* (category G, 8.42% of assignments).

Since the absence of antimicrobial resistance is a very important trait in potential probiotics (Binda et al., 2020), the prediction of this feature was conducted by three independent strategies, one based on mapping against NDARO, other comparing against Resfinder and a third one based on RGI-CARD. According to NDARO and Resfinder, no antimicrobial resistance marker was predicted. However, predictions from RGI-CARD showed two classes of antimicrobial resistance: an intrinsic resistance to mupirocin, given by *ileS* alleles (ARO:3003730, 99.46% identity, 100.09% coverage) and an intrinsic resistance to rifampicin, given by *rpoB* alleles (ARO:3004480, 91.22% identity, 100.08% coverage). Those two resistance markers were driven by mutations previously reported in *Bifidobacterium* species (Serafini et al., 2011; Lokesh et al., 2018). In contrast, specialized enzymes or acquired markers were not found in *B. bifidum* 900791. These results may suggest that this strain can be suitable for its use as a probiotic.

### 3.2 The 900791 strain gene content in the context of the *B. bifidum* pangenome

In order to obtain a better picture of the predicted functionality of the 900791 strain, we also compared the predicted gene content of this strain with a set of 228 *B. bifidum* genomes, to obtain the pangenome of this species. According to metadata search (**Supplementary Table 1**), most assemblies were obtained from reported human samples (214 strains, 93% of the total). More than 79% of the selected assemblies were obtained from isolated strains. Only 30 metagenome-assembled genomes (MAGs) were found. Also, most strains were reported to be obtained from stool (60% of the total), several others without clear metadata (e.g., mentioning that they are from faecal samples but not establishing whether they are from adults or children), and only a few are from other sources such as bladder, fermented milk, rumen or vaginal samples.

**Figure 1:**
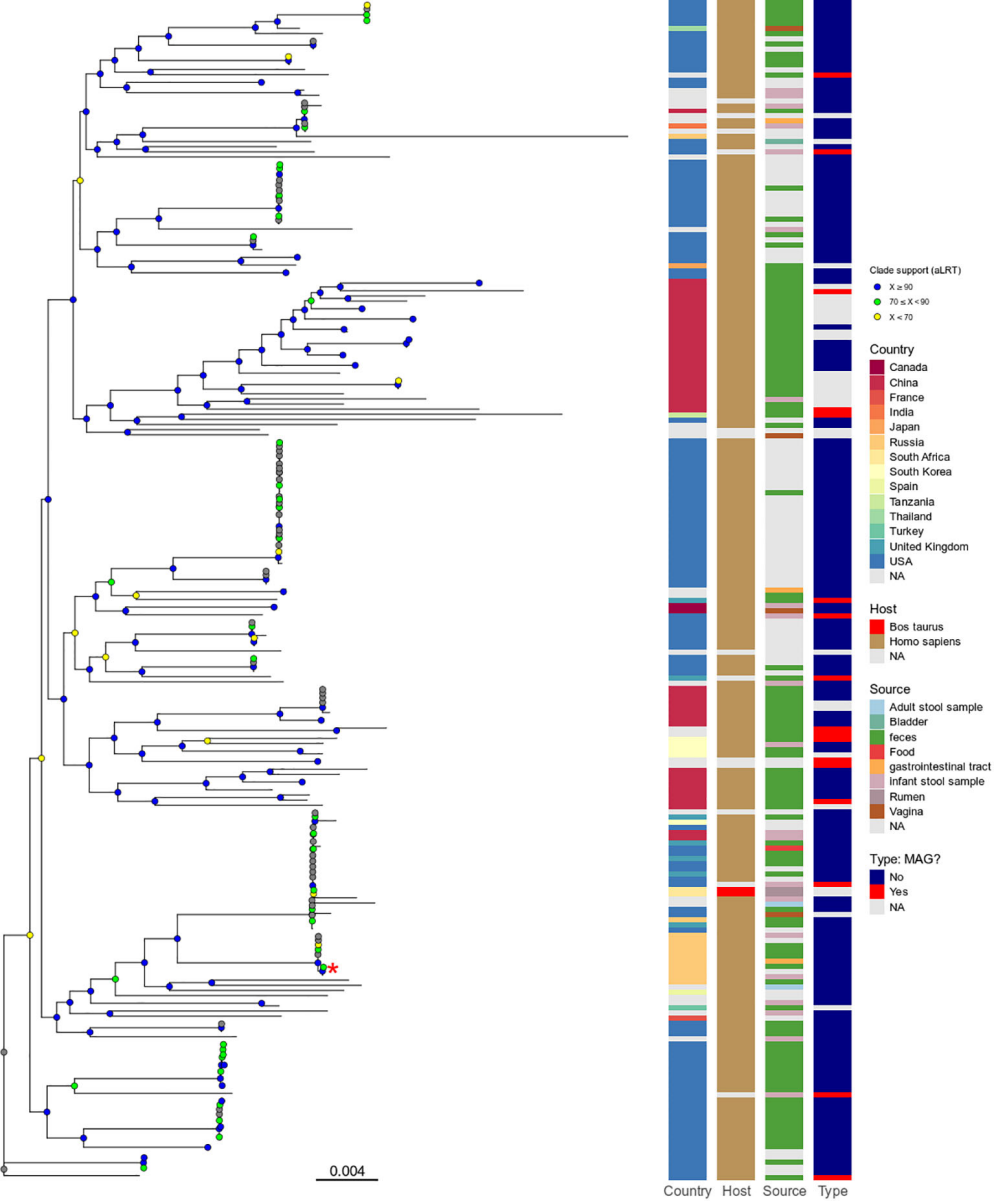
Phylogenomic tree showing the relationships between different *Bifidobacterium bifidum* strains. The tree was created from the alignment of 769 single-copy conserved protein families, using the maximum likelihood method in IQTREE (“-m TEST” mode), with the use of the approximate likelihood ratio test (aLRT) as the branch support test. The outgroup (the proteome of *B. longum* subsp. *longum* JCM 1217) was removed to improve branch resolution. Colored nodes indicate different branch support values ranges. Columns from the right side represent metadata fields reported by the respective NCBI Assembly/Biosample accession. The branch with the red asterisk (“*”) is where the 900791 strain is located.

In order to understand the evolutionary dynamics of the *B. bifidum* 900791 evolution, we performed phylogenetic trees from two protein- and nucleotide-based approaches. The amino acid-based phylogenomic tree was elaborated from the Orthofinder-defined single-copy orthologs (**Figure 1**). On the other hand, the codon-adjusted nucleotide alignment for core gene families obtained from Panaroo was used for a second phylogenomic tree (**Supplementary Figure 3**). Cophylogeny analysis suggests important differences between those two trees, although most external clades had relatively low (aLRT < 75) branch support values (**Supplementary Figure 3**). In accordance with this observation, a RF distance of 0.64 indicates that nearly 64% of the branch splits are different between trees, suggesting extensive topological differences. However, the KF distance, a measure for the difference between branch lengths of shared splits, was quite low (KF = 0.052), suggesting that most branch lengths observed between trees were nearly identical. In order to elucidate the best explanation for this combination of RF and KF values, a RF was performed only considering branches with branches with length higher than 0.01, giving a value of 1; this RF value suggests that similarities between tree topologies were derived virtually from short branches only. This feature has concordance with the abundant number of branches with low support values, suggesting that members of the species are similar enough to have clade resolution problems.

Although this strong low-distance phylogenetic relationships, both trees showed that the 900791 strain was strongly related to other eight genomes, belonging to the strains 791 (GCA_001595435, GCA_022014355), ICIS-310 (GCA_002114145), ICIS-643 (GCA_003790385), ICIS-202 (GCA_004799295), VKPM Ac-1784 (GCA_016070035), ICIS-629 (GCA_020710245), and ICIS-176 (GCA_020861855), all of them isolated from human stool samples from Russian subjects (**Supplementary Table 1**). Moreover, the results of the cgMLST suggested that 900791 and the other eight related genomes contained practically the same alleles (>99%) as each other, in comparison to the rest of the genomes. This phylogenetic pattern and allele content suggest that strain 900791 could be part of a clonal sublineage of *B. bifidum*, with potentially distinct genomic features.

**Figure 2:**
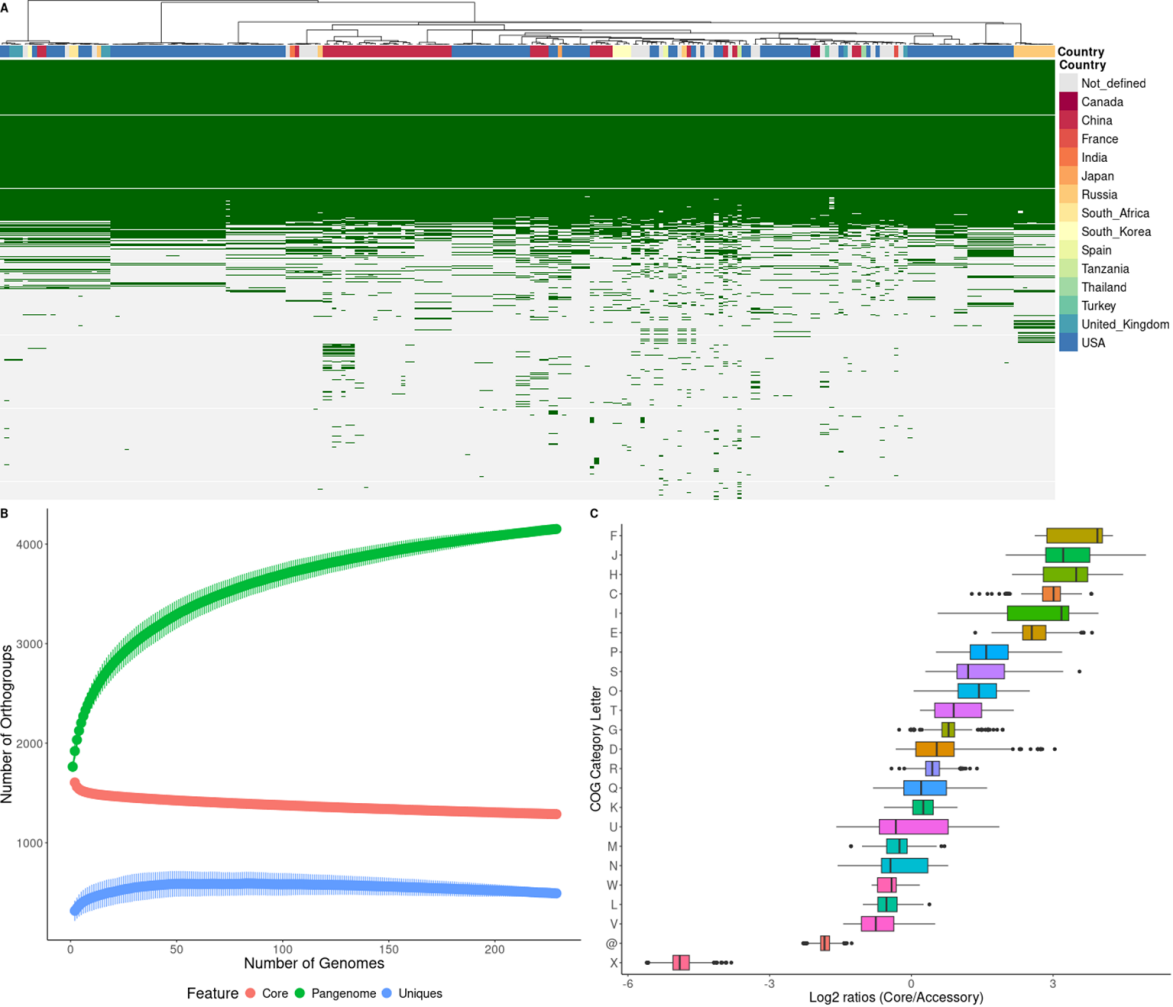
Pangenome analysis of *B. bifidum* utilizing 229 high-quality genomes (including the 900791 strain). **(A)** Ortholog conservation heatmap for the genomes according to their geographical origin, clusterized their shared gene family profile. **(B)** Pangenome accumulation curve suggests that the pangenome for *B. bifidum* is open (gamma value = 0.16, according to Power Law equation). **(C)** Analysis for the Log2 ratios of the percentage of core/near-core (>= 95% prevalence) versus accessory (< 95% prevalence) genes for each COG category among pangenomes “(@” = no COG category). COG category descriptions are available on the *COG Database website* (https://www.ncbi.nlm.nih.gov/research/cog).

In order to gain more insights about the gene content of these species, we made a pangenome analysis using the ortholog matrix produced from Panaroo results, in combination with other tools, to interrogate different aspects of the *B. bifidum* pangenome (**Figure 2**). The analysis of a total of 414, 526 CDS showed that the pangenome of this species is composed of 4, 152 orthogroups, 491 of them represented by unique genes. The core orthogroup set (i.e., the set of ortholog families present in all 229 genomes) contained 1, 450 families. Panaroo data suggests that no orthogroup was unique in the 900791 strain in comparison with the other 228 genomes; interestingly, 900791 shared 23 orthogroups exclusively with the eight genomes from the aforementioned clonal subgroup strains, encoding several ORFans, membrane proteins and a potential lantibiotic biosynthetic cluster (**Supplementary Table 2**); lantibiotics are well-known compounds that function as antibiotics, causing antimicrobial effects by either forming lethal membrane pores or via inhibition of peptidoglycan biosynthesis (Brötz and Sahl, 2000). The clustering pattern in orthogroup heatmap (**Figure 2A**) suggests that the same genomes related to 900791 strain were also related in terms of orthogroup content, increasing the possibility of a clonal subgroup (see above). The pangenome accumulation curve (**Figure 2B**) showed a power law equation (*y = ax^γ^*) with a gamma value of 0.16251; alternatively, the alpha value from the Heap’s law, also utilized to define the openness of a pangenome, was equal to 0.825. Those values indicate that the B. *bifidum* pangenome, although being open, exhibits a curve with a slow expansion, indicating limited available genomic plasticity. When the functional annotation for the core/near-core (i.e., gene families with >= 95% prevalence among genomes) gene fraction was compared with the accessory gene fraction (i.e., families with < 95% prevalence), a well-defined conservation profile was obtained (**Figure 2C**): functions associated to information transfer (categories J: “Translation, ribosomal structure and biogenesis”) and metabolism (categories F: “Nucleotide transport and metabolism”, C: “Energy production and conversion”, H: “Coenzyme transport and metabolism”, I: “Lipid transport and metabolism”, and E: “Amino acid transport and metabolism”) were found with a high Log2 ratio for the core-vs-accessory fractions, suggesting that those kind of functions have been selected to belong to the core genome of this species. Additionally, the strong presence of translation-associated genes in the core fraction, such as the ones involved in the ribosome assembly and structure, may reflect how these genes are recalcitrant to be transferred horizontally, making them less susceptible to be present in the accessory fraction (Kanhere and Vingron, 2009). In counterpart, genes in categories X: “Mobilome: prophages, transposons” and V: “Defense mechanisms”, as well as genes without COG (see the ‘@’ boxplot in **Figure 2C**), were found with the more negative Log2 ratios in the core-vs-accessory comparison. The presence of this kind of functions, as well as the enrichment of ORFans in the accessory pangenome, as potential main contributors to the openness of a pangenome, are already well-documented phenomena (e.g (Rajput et al., 2023)).

**Figure 3:**
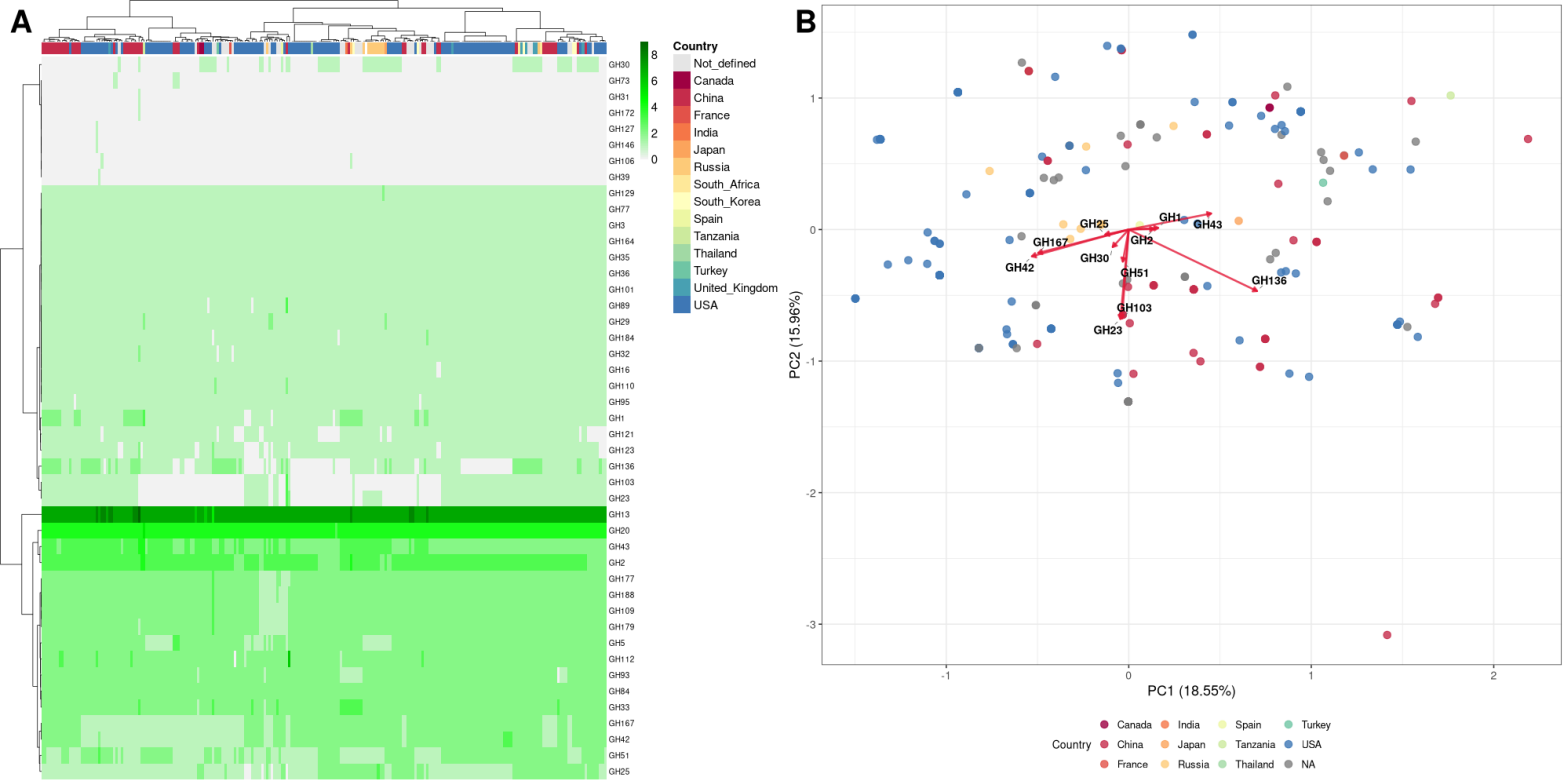
Comparative analysis of GHs profile of *B. bifidum* utilizing 229 high-quality genomes (including the 900791 strain), characterized by country of origin. HMM profiles from the dbCAN version 15 were used against the predicted CDS of 229 *B. bifidum* strains and clusterized (by euclidean distances) in a heatmap **(A)** and in a PCA dotplot showing the most important loadings influencing the dispersion of the genomes by their GH content **(B)**.

A relevant feature expected to be found in *B. bifidum* strains is the presence of genes involved in the degradation of a variety of glycans and fibers. *B. bifidum* strains harbor a variety of GHs (**Figure 3**). CAZyme profiling using dbCAN-CAZy HMM profiles revealed that the genome of strain 900791 encodes representatives of GH families previously associated with HMO degradation ((Kitaoka, 2012), GH2, GH20, GH33, GH84), mucin glycan cleavage ((Lordan et al., 2024), GH33, GH29, GH95, GH20, GH2, GH42, GH101, GH129, GH89, GH84), and lactose utilization ((Ambrogi et al., 2019), GH2, GH42) (**Figure 3A**). This enzymatic repertoire is consistent with the previously reported ability of this strain to improve lactose tolerance in human subjects (Aguilera et al., 2021). GH-content-based clustering also placed 900791 within the clonal subgroup, confirming that the characteristic CAZyme profile is shared across this geography-specific lineage (**Figure 3A, B**); the GH families most responsible for subgroup-specific clustering were GH43, GH2, GH51, GH25, GH1, GH136, GH167, GH42, GH103, GH23, and GH30 (**Figure 3B**).

### 3.3 Predicted probiotic features of the 900791 strain and its clonal subgroup

As seen previously, the 900791 strain is related to other *B. bifidum* strains forming a potentially clonal group. Since this strain is an interesting probiotic candidate, it is necessary to track such potential features in its genome to predict its success and utility as a probiotic agent. Genomic information available for different probiotics are a valuable source for the search and identification of genotypic traits that ensure efficacy, safety, and stability. Strain-specific genetic variability requires a precise understanding of conserved and accessory genes to optimize selection criteria. According to previously studied models, some important markers for probiotic properties include genes involved in adhesion to intestinal mucosa, polysaccharide synthesis, and substrate utilization, which are essential for gut colonization and persistence (Rossi et al., 2022). Stress factors involved in low pH and bile salt resistance are also important features to look for in probiotics (Ruiz et al., 2013; Collado and Sanz, 2007). Additionally, other desirable traits are the verified absence of antimicrobial resistance markers and virulence factors (Li et al., 2024b) and the ability to exhibit a proper oxidative stress response (Feng and Wang, 2020). In order to establish the predicted features of the 900791 strain and its related strains, we will separate this section into different features:

#### 3.3.1 Adhesion to mucin and epithelial cells

**Figure 4:**
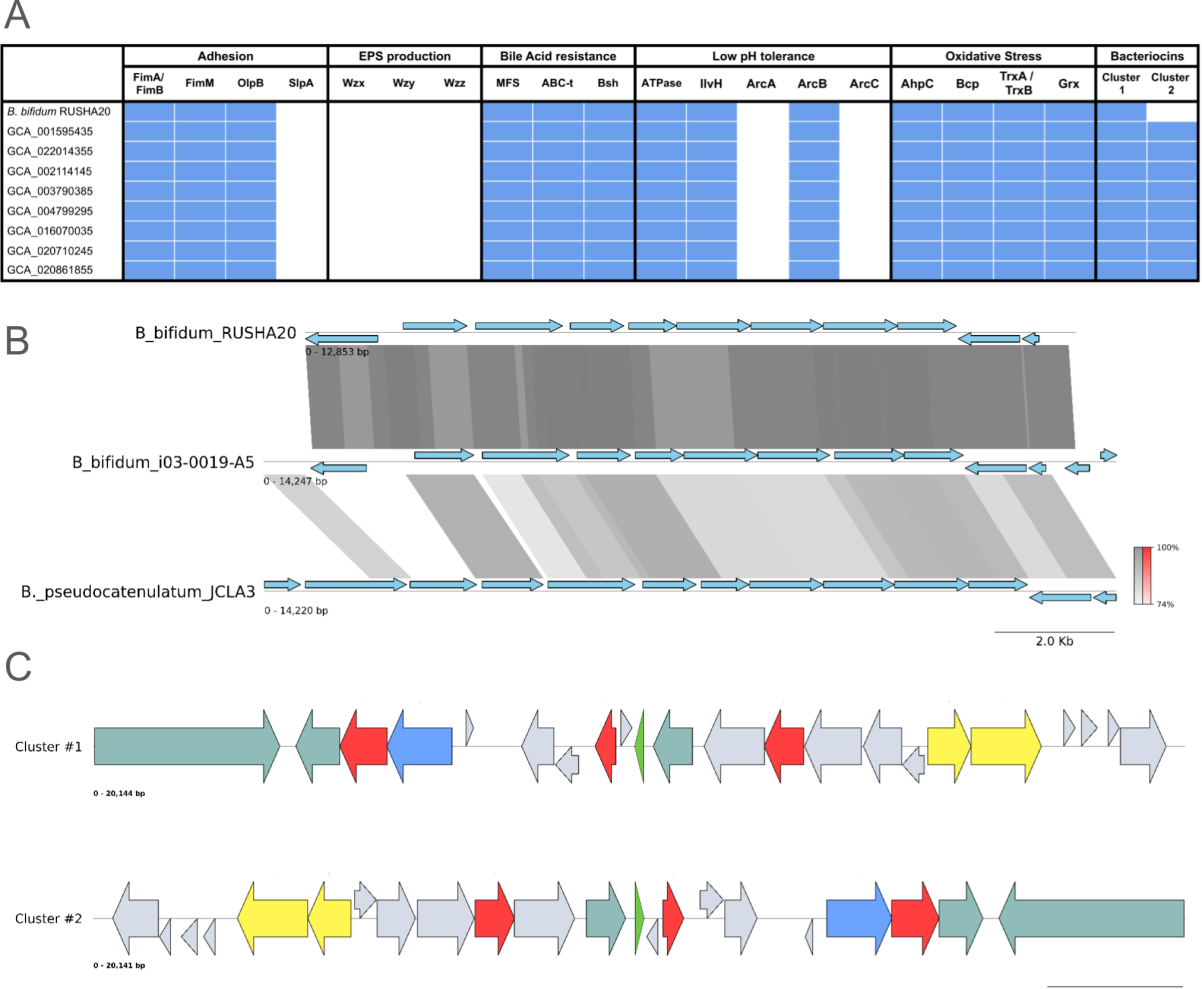
Conservation of genes involved in different predicted probiotic traits in strain 900791 and related strains. **(A)** table of genetic features searched for in the characterization of the 900791 strain and the other members of the clonal group. **(B)** Conservation of the closest gene cluster potentially involved in EPS biosynthesis. **(C)** features of the found clusters/AOIs involved in bacteriocin biosynthesis, showing relevant proposed roles in different colors; Green: bacteriocin-encoding gene; red: genes encoding components of ABC-type transporters; yellow: potential regulators, dark green: potential multidrug exporting systems; blue: genes involved in lantibiotic biosynthesis; grey: other functions.

The genome of strain 900791 and all eight related clonal strains encode at least three orthogroups belonging to COG4932, the family that includes the pilus-forming proteins FimA/FimB and the mucin-adhesin FimM (**Figure 4A**). This repertoire of adhesion-associated proteins suggests that strain 900791 retains the cell-binding machinery characterized in other *B. bifidum* strains (Xiong et al., 2020; Milani et al., 2017). Additionally, *in-silico* prediction identified a gene encoding a COG1361 family member [related to the cellulosomal surface protein OlpB from *Clostridium thermocellum* (Fujino et al., 1993) in 900791 and its clonal group; we hypothesize that this protein may perform an S-layer-like adhesive function analogous to SlpA in *Lactobacillus* (Frece et al., 2005; Prado Acosta et al., 2016), although experimental validation is required (**Figure 4A**).

#### 3.3.2 Exopolysaccharide production

Analysis of the 900791 genome and its clonal group identified a single candidate gene cluster flanked by transposases encoding putative EPS precursor biosynthesis enzymes, glycosyltransferases, and an ABC transport system (**Figure 4B**) related to a previous eps cluster found in other *Bifidobacterium* strains (Ferrario et al., 2016). This cluster shares >99% nucleotide identity with a cluster in *B. bifidum* i03-0019-A5 (accession AP031420) and ~74% identity with a cluster in *Bifidobacterium pseudocatenulatum* JCLA3 (accession CP090598). Notably, no candidates for the key export-polymerization components (flippase Wzx, polymerase Wzy, chain-length determination protein) were identified in 900791 or its clonal group, even when searches were extended using previously reported sequences from other bifidobacteria (Ferrario et al., 2016). The absence of these components suggests that strain 900791 may lack functional EPS export machinery, and consequently may not produce surface EPS; this prediction requires experimental confirmation.

#### 3.3.3 Bacteriocin production

Another important feature to be observed in probiotics is the ability to produce bacteriocins, peptides that are active against other bacteria without causing effect on the producing microorganism (Anjana and Tiwari, 2022). BAGEL analysis of the 900791 genome and its clonal group identified two bacteriocin-associated gene clusters marked as “areas of interest” (AOIs). Cluster/AOI #1, present in 900791 and all eight related strains, encodes a putative lanthipeptide A bacteriocin of the *Geobacillin_I_like* family; members of this group exerted previously verified antimicrobial activity ((Garg et al., 2012); **Figure 4C**). Cluster/AOI #2 was absent from 900791 itself but was found in the eight other clonal group members; it encodes a putative *Lasso peptide* of the *Propeptin_2* family, with similarity to a peptide from *Microbispora* sp. SNA-115 that inhibits prolyl endopeptidases and shows activity against *Mycobacterium phlei* (Kimura et al., 2007). Both clusters contain genes encoding ABC transporter components and lantibiotic biosynthesis domains (PF05147, PF00733), supporting their functional annotation as bacteriocin loci with potential impact on other bacterial species.

#### 3.3.4 Acid (low pH) resistance

Another desirable trait in potential probiotic microorganisms is the ability to tolerate low pH, since it is required for survival in their passage through the acidic gastric environment (Huys et al., 2013) (Jin et al., 2015, 2012; Huys et al., 2013). All nine strains in the 900791 clonal group encode a complete set of genes for the F_0_F_1_-ATPase system (*atp* genes) involved in proton extrusion (Sánchez et al., 2007), as well as a copy of the IlvE branched-chain-amino-acid transaminase (EC 2.6.1.42; trEMBL accession I3WHR6), an enzyme implicated in low-pH metabolic responses in both *Streptococcus mutan*s (Len et al., 2004) and *Bifidobacterium longum* (Sánchez et al., 2007) (**Figure 4A**). This analysis found a putative copy encoding ArcB (ornithine carbamoyltransferase; trEMBL O53089), part of the *Lactobacillus sakei* arginine deimination pathway, also induced on low pH (Zúñiga et al., 1998; Rimaux et al., 2012); no ArcA (arginine deiminase) or ArcC (carbamate kinase) candidates were detected, suggesting this three-step ammonia-releasing pathway is not utilized by this clonal group.

#### 3.3.5 Bile salt resistance

Probiotic strains have the ability to tolerate bile salts, common stress-inducing factors that microorganisms must face in the gastrointestinal tract in their colonization or passage process (Huys et al., 2013). The genome of strain 900791 and its clonal group encode orthologs of the MFS transporter Bbr_0838 (Uniprot accession C4NXS7_BIFBU, previously characterized in *B. breve* UCC2003 as a bile salt resistance determinant (Ruiz et al., 2012a)), the ABC transporter subunits Bbr_0406–0407 and Bbr_1804-1805 (also involved in bile salt stress response (Ruiz et al., 2012b)), and the bile salt hydrolase Bsh (Tanaka et al., 2000); **Figure 4A**). Together, these findings indicate that the 900791 clonal group possesses both enzymatic (BSH-mediated deconjugation) and efflux-based (MFS and ABC transporter) strategies for bile salt resistance.

#### 3.3.6 Oxidative stress

Since probiotics need the ability to survive during industrial production and storage, as well as their transit through the gastrointestinal tract, the response to sudden changes in reactive oxygen species (ROS) is key in their successful maintenance, passage and survival (Blazheva et al., 2022; Schöpping et al., 2022; Imlay, 2003). The 900791 strain and its clonal group encode a core set of oxidative stress response proteins, including the AhpC peroxiredoxin (COG0450), glutaredoxin (Grx, COG0695), peroxiredoxin Bcp (COG1225), thioredoxin (TrxA, COG0526), and thioredoxin reductase (TrxB, COG0492) (**Figure 4A**). This repertoire suggests that strain 900791 is equipped to scavenge reactive oxygen species and maintain redox homeostasis under oxidative conditions encountered during industrial processing, storage, and gastrointestinal transit.

## 4 Discussion

In this study, we analyzed the complete genome of *Bifidobacterium bifidum* strain 900791 and we compared its CDS repertoire with the content of 228 other *B. bifidum* genomes Although this study is exclusively *in-silico*, several important insights emerge regarding its potential as a probiotic candidate and its distinctive genomic characteristics. The genomic characteristics of strain 900791 are consistent with typical *B. bifidum;* for example, its genome size, number of ORFs and %G+C fall within the expected range for *B. bifidum*, as previous studies have reported: genome sizes: 2.03–2.55 Mb (average = 2.17 ± 0.09 Mb); average of ORFs per genome: 1837 ± 143; G+C content ranging between 62.3% and 62.8% (Lu et al., 2021). The functional annotation reveals a genome adapted for gut colonization and carbohydrate metabolism, hallmarks of successful gut commensals, seen commonly in other members of the species (Glover et al., 2022; Raba and Luis, 2023). The presence of a rich variety of GHs from families associated with degradation of HMOs and mucin glycans, including GH2, GH20, GH33, and GH84, underscores the capacity of this *B. bifidum* strain for utilizing host-derived glycans, in correspondence with the fact that this strain was obtained from human feces. Additionally, the detection of GH2 and GH42 families specifically associated with lactose degradation provides genomic support for the previously observed clinical improvement in lactose tolerance in human subjects consuming this strain, suggesting the use of lactose as a validated trait (Aguilera et al., 2021). The subcellular localization predictions indicating that 28.2% of proteins are located outside the cytoplasm suggest an active secretome and membrane-associated machinery that may reflect the great importance of host-microbe interactions for the physiology of this organism, covering secretable CAZymes, active proteins and other components influencing the interaction with the host-associated environment (Vidal-Veuthey et al., 2022). Thus, this extensive repertoire of exposed proteins is consistent with the lifestyle of gut-adapted bifidobacteria, which must interface effectively with the host intestinal environment.

This study, as far as we know, also presented the largest *B. bifidum* pangenome analysis to date. The detection of a slightly open pangenome structure, with slow gene expansion, may indicate that the need for genome plasticity required for adaptation in the human gut is starting to require fewer gene families. This feature may highlight the strong adaptation of *B. bifidum* to become a niche specialist (Brockhurst et al., 2019) that has largely converged on its optimal gene repertoire, yet continues to sample fewer and fewer adaptive innovations from the global diversity of the human gut ecosystem (Shoer et al., 2024).

Our study detected a pangenome of 4, 152 orthogroups and a genomic core of 1, 450 gene families; these values are quite different from those reported by another study (Lu et al., 2021) showing a pan-genome of 8, 399 genes and a core genome of 638 genes obtained from a set of 140 assemblies. However, this same study showed that a subset of 55 reference genomes obtained a pan-genome of 4, 602 genes, closer to our results. Regardless of the properties of the assemblies used in the other study, our higher core genome can be explained by our requirement of higher assembly contiguity and high completeness (N50 > 200 Kbp plus >=90% completeness values), in comparison with a wider range of N50 values used in the Lu et al., genome dataset. Previous studies have suggested that higher contiguity may help to recover a higher core genome is associated with better assembly contiguity and completeness (Vollmers et al., 2022). Functional enrichment patterns observed in the core fraction are completely expected: genes involved in translation, amino acid metabolism, and energy production, reflect the essential cellular processes required for survival. The overrepresentation of accessory genes in mobilome and defense functions indicates that these represent major sources of genomic variation and adaptation within the species; this observation was also made in other bacterial organisms (e.g., (Liao et al., 2023)).

The phylogenomic analyses, in combination with cgMSLT analysis, showed that 900791 belongs to a distinct clonal subgroup comprising nine closely related strains, all isolated from human stool samples (mostly from children). This clonal relationship, supported by >99% allelic similarity in cgMLST analysis, suggests recent common ancestry and shared adaptive strategies; also, the geographical clustering of this clonal group was relevant (all strains came from the same country, see **Figure 1**); these patterns suggest that possible population structure within *B. bifidum* may reflect local adaptation or founder effects. Moreover, the identification of 23 unique orthogroups exclusive to this clonal subgroup (including a set of genes putatively involved in lantibiotic biosynthesis), reinforces the importance of these strain-specific adaptations.

This study provided the *in-silico* characterization of the potential use of the 900791 strain as a probiotic, showing that this strain contains a genome encoding several predicted traits for probiotic use. The safety profile of 900791 represents a pivotal foundation for the justification of its probiotic potential. The comprehensive antibiotic resistance analysis using three independent methods (NDARO, ResFinder, and RGI-CARD) revealed only intrinsic resistance to mupirocin and rifampicin, mediated by naturally occurring mutations in *ileS* and *rpoB* genes respectively. These resistance markers are well-documented common mutations in bifidobacteria and those strains have been used as probiotics regardless of the presence of those resistance markers (Grmanová et al., 2010; Serafini et al., 2011). Since determinants for acquired or transferrable antimicrobial resistance genes, plasmids, and virulence factors were not found, it is possible to suggest that this strain has a safe antimicrobial resistance profile for a probiotic (Perunova et al., 2025).

The genomic analysis also revealed multiple stress tolerance systems that are essential or desirable for probiotic functionality: genes involved in the tolerance to low pH experienced during gastric passage (e.g., by the presence of the F_0_F_1_-ATPase systems or a branched-chain amino acid transaminase); genes involved in the extrusion or conversion of bile salts to improve the tolerance to those compounds in the passage through the small intestine (Bbr_0838-like MFS transporters, ABC transporter subunits, and the Bsh hydrolase); or the potential ability to tolerate fluctuant exposition to ROS via a proper oxidative stress response (via enzymes such as thioredoxin-thioredoxin reductase systems, alkyl hydroperoxide reductase, peroxiredoxins, glutaredoxins). All those stress responses predicted in the genome of the 900791 strain and conserved in the rest of its clonal group may suggest that this strain may be well equipped for the challenges that its use as a probiotic requires.

The genomic repertoire of 900791 also included several factors associated with beneficial host-microbe interactions: the identification of genes encoding FimA/FimB and FimM, as well as a putative S-layer protein, indicates the potential of this strain to interact with mucin or epithelial cells, in order to improve colonization efficiency, another desired feature for probiotic bacteria. Additionally, bacteriocin production capability of 900791 (and also of the other strains of their clonal group) represents a significant competitive advantage in the complex gut ecosystem. This potential antimicrobial capacity may contribute to the ability of 900791 to suppress pathogenic bacteria, a desirable probiotic trait (Yu et al., 2023).

Finally, whereas genomic analysis provides strong evidence for the probiotic potential of the 900791 strain, experimental validations of the predicted stress tolerance mechanisms under physiologically relevant conditions, or for the other features, are key aspects to investigate in future work. *In-vitro* (and further *in-vivo*) studies evaluating colonization dynamics, persistence, and interaction with the existing gut microbiota would provide crucial insights into the key features of the 900791 phenotype as a candidate probiotic. Experimental validation of the genomic predictions reported in this study is the mandatory further step, including *in-vitro* adhesion assays to mucin and/or intestinal epithelial cell lines, acid- and bile salt survival assays at physiologically relevant concentrations and antimicrobial activity assays. Moreover, additional clinical trials evaluating specific health benefits beyond lactose tolerance, such as immune modulation or metabolic control, are also important aspects to investigate in order to gain a better insight of the therapeutic potential of this strain. Experimental, comparative studies with other members of the clonal subgroup could also reveal whether the shared genomic features translate to similar probiotic properties, potentially identifying a group of related strains with complementary or enhanced therapeutic applications.

## Data Availability

The datasets presented in this study can be found in online repositories. The names of the repository/repositories and accession number(s) can be found in the article/**Supplementary Material**. The raw read data for *B. bifidum* 900791 utilized in this study is available in the NCBI Sequence Read Archive under submission accession number PRJNA1375603.

## Supplementary material

Supplementary Table 1 List of the 228 *B. bifidum* genomes retrieved from NCBI and selected for this study, including information from GTDB and CheckM2 results, and metadata retrieval.

Supplementary Table 2 List of the functional annotation of the set of 23 orthogroups exclusively conserved in the clonal group related to the 900791 strain.

Supplementary Figure 1 Circular representation of the complete genome of the 900791 strain including genomic features and predicted functions according to COGs.

Supplementary Figure 2 Main annotation stats for the 900791 strain annotated CDS. **(A)** Piechart showing the predicted subcellular localization according to PSORTb 3.0 prediction (Gram-positive mode). **(B)** Piechart showing the distribution of the COG metacategories according to EGGNOG mapper predictions. **(C)** barplot showing the represented COG categories among the predicted CDS.

Supplementary Figure 3 Tanglegram comparing the amino acid- (left) and nucleotide-based (right) phylogenies of the 229 *B. bifidum* genomes. Branch support (BS) values were displayed as node colors. Red: BS < 75, Yellow: 75 < BS < 95, Green: BS ≥ 95.
