# Supplementary figures and images for: In-silico analysis of Bifidobacterium bifidum strain 900791 genome in the context of the B. bifidum pangenome
Source: Front Cell Infect Microbiol. 2026 Jul 1;16:1744409. doi: 10.3389/fcimb.2026.1744409 (PMC13368793; doi:10.3389/fcimb.2026.1744409)

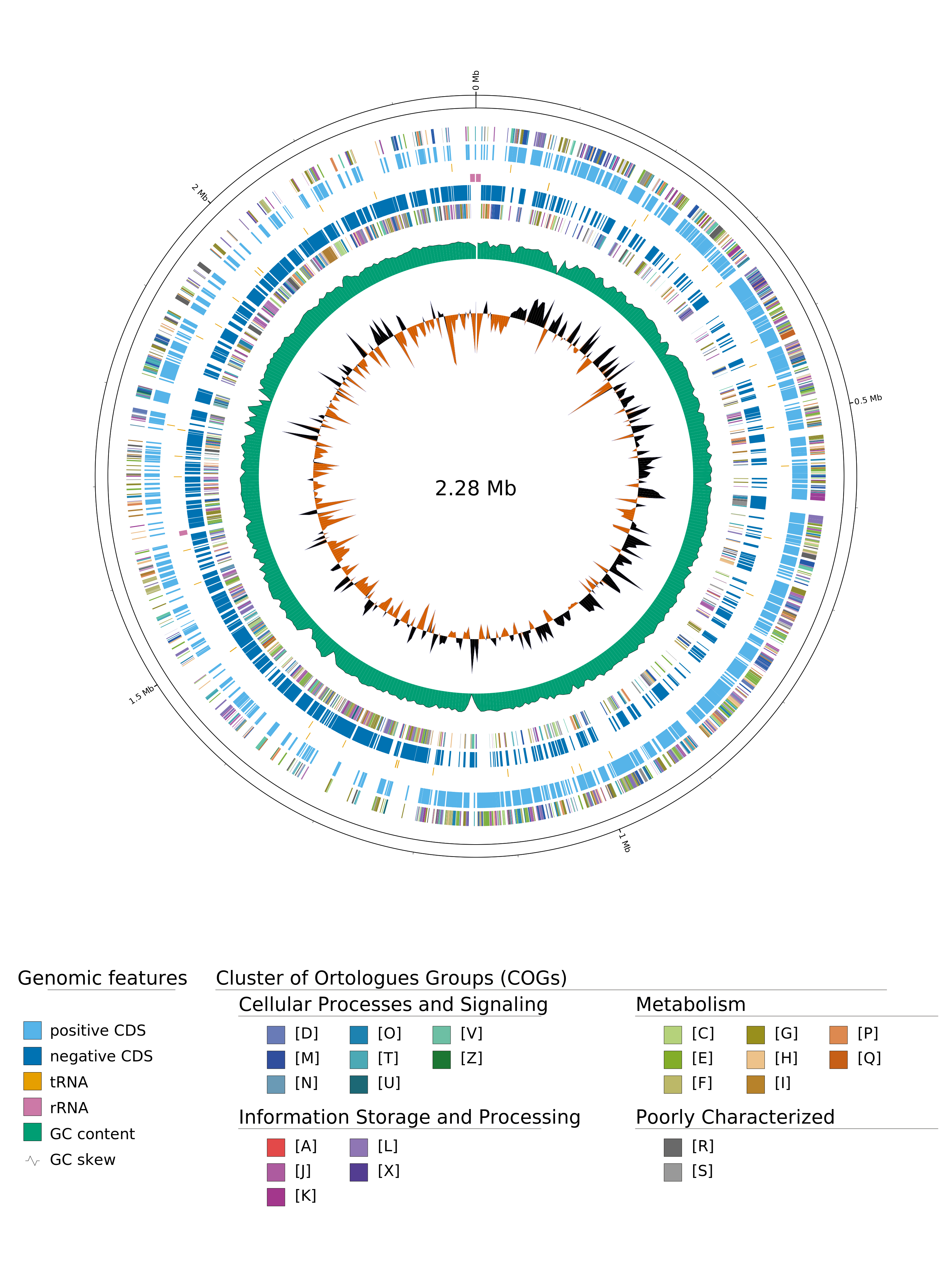

Supplement: Supplementary Figure 1 — Circular representation of the complete genome of the 900791 strain including genomic features and predicted functions according to COGs. [file Image1.png]

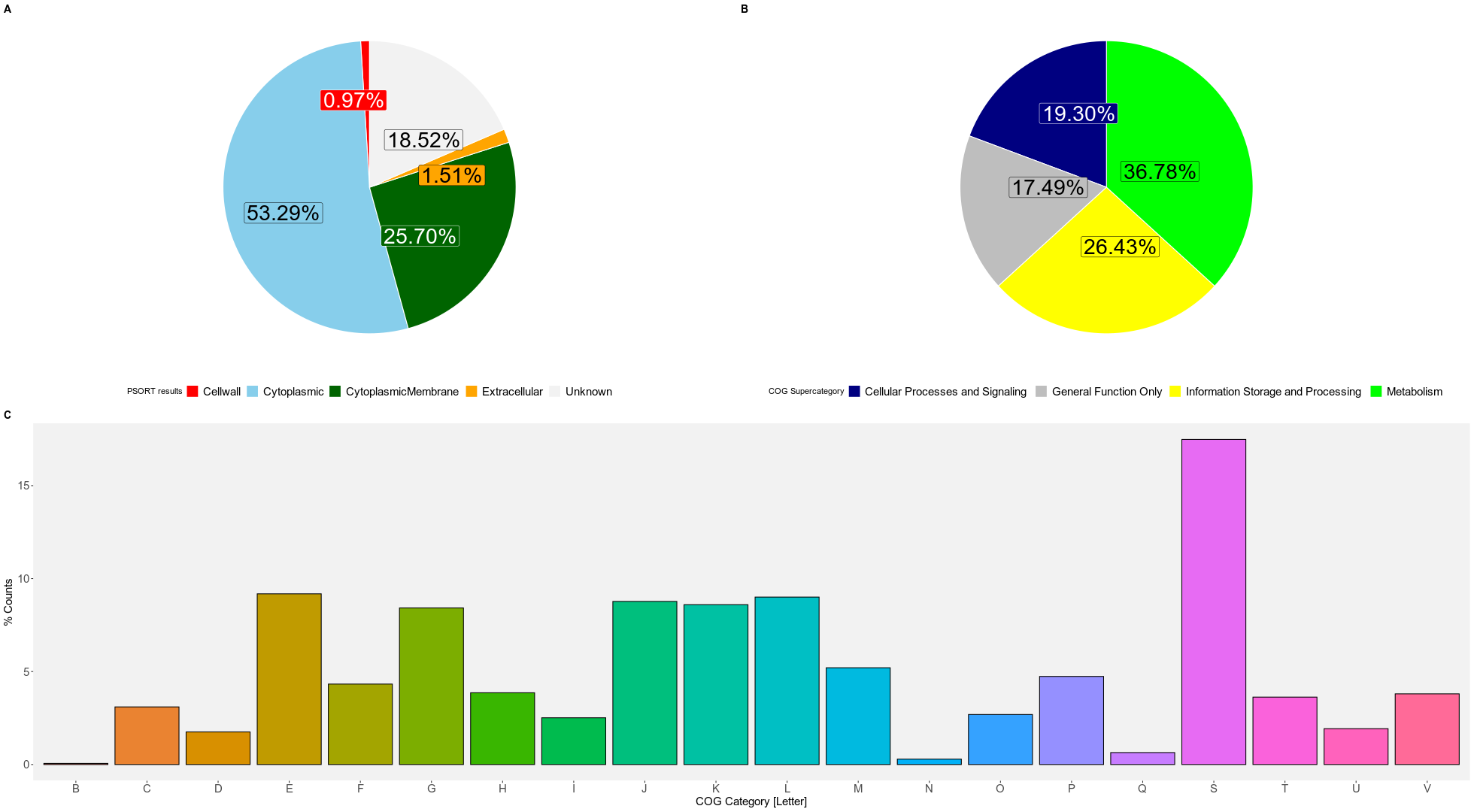

Supplement: Supplementary Figure 2 — Main annotation stats for the 900791 strain annotated CDS. (A) Piechart showing the predicted subcellular localization according to PSORTb 3.0 prediction (Gram-positive mode). (B) Piechart showing the distribution of the COG metacategories according to EGGNOG mapper predictions. (C) barplot showing the represented COG categories among the predicted CDS. [file Image2.png]

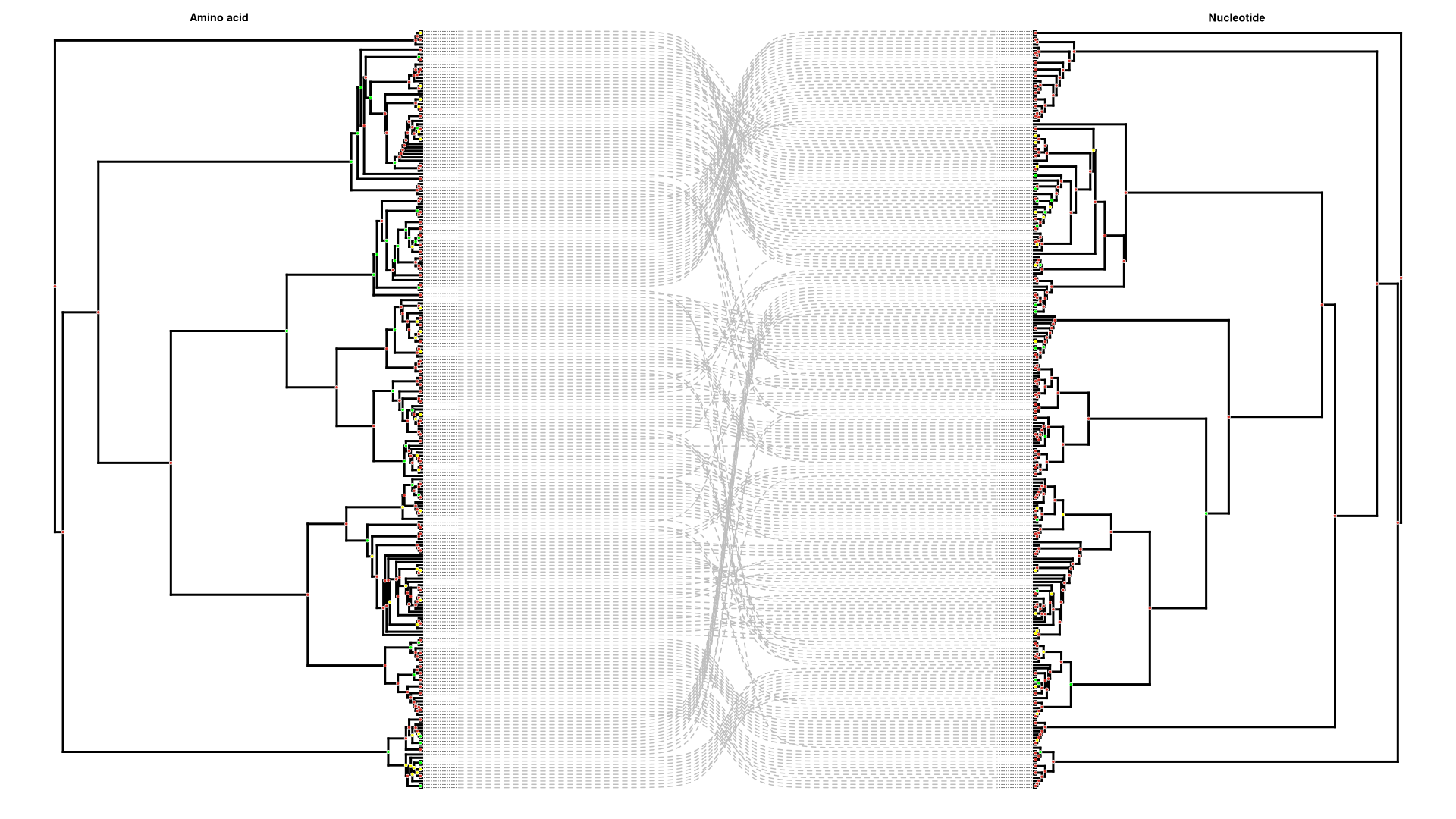

Supplement: Supplementary Figure 3 — Tanglegram comparing the amino acid- (left) and nucleotide-based (right) phylogenies of the 229 B. bifidum genomes. Branch support (BS) values were displayed as node colors. Red: BS < 75, Yellow: 75 < BS < 95, Green: BS ≥ 95. [file Image3.png]
